# Secondary peritonitis -evaluation of 204 cases and literature review

**Published:** 2014-06-25

**Authors:** SK Doklestić, DD Bajec, RV Djukić, V Bumbaširević, AD Detanac, SD Detanac, M Bracanović, RA Karamarković

**Affiliations:** * Clinic for Emergency Surgery, Clinical Center of Serbia, Belgrade, Serbia; ** School of Medicine, University of Belgrade, Belgrade, Serbia; Clinic for Emergency Surgery, Clinical Center of Serbia, Belgrade, Serbia; *** School of Medicine, University of Belgrade, Belgrade, Serbia; Department of Anesthesiology, Clinic for Emergency Surgery, Clinical Center of Serbia, Belgrade, Serbia; **** Health Center Novi Pazar, Serbia

**Keywords:** secondary peritonitis, acute abdomen, emergency surgery

## Abstract

Abstract

Hypothesis: Even at the beginning of the new millennium, secondary peritonitis presents a common life-threatening condition associated with high mortality and morbidity.

Objective: This article comments on epidemiology, diagnosis and general principles of surgical management in patients with secondary peritonitis.

Methods and Results: The demographic data, clinical findings and surgical outcome of 204 patients who had a confirmed generalized secondary peritonitis were analyzed retrospectively. Our approach was laparotomy, surgical control of contamination, antibiotic therapy and modern intensive care support. Acid peptic disease was the most common cause of perforation peritonitis 60 (29,41%), following by the perforated appendicitis 45 ( 22,06%). The faecal peritonitis and colon perforation were found in 42 patients (20,59%). The morbidity rate was 50%; 41 (40,2%) patients had more than one complication. The morbidity rate was significantly the highest in patients with colon perforation (n=38, 90%) (Hi-square=40,1; p<0,001). The overall mortality rate in our study was 8,82%. The mortality rate was significantly the highest among the patients with mesenteric ischemia in 4 patients (66,67%), followed by colon perforation in 10 cases (23,81%), and 4(6,6%) deaths due to gastro-duodenal perforation (Hi-square=45,7; p<0,001).

Discussion: This study has confirmed that the clinical presentation and outcome of the secondary peritonitis depend on duration of abdominal infection, the site of perforation and the general condition of the patient. Rapid surgical source control, modern intensive care and sepsis therapy may offer the chance of decreased morbidity and mortality of the intra-abdominal infections.

Abbreviations

Intensive Care Unit (ICU), Perforated Diverticular Disease (PDD), Inflammatory Bowel Disease (IBD), Multiple Organ Dysfunction Syndrome (MODS), Acute Respiratory Distress Syndrome (ARDS), Nonsteroidal Anti-inflammatory Drugs (NSAID), Peptic Ulcer Disease(PUD), Ultrasonography (US), Computer Tomography (CT), Colorectal Cancer (CRC), Postoperative Adhesive Disease (PAD), Acute Mesenteric Ischemia (AMI).

## Introduction

Generalized secondary peritonitis is one of the most common surgical emergencies. Despite the great progress in intensive care support, antimicrobial therapy and surgical techniques, the management of peritonitis is still highly complex and represents a challenge for clinicians [**1**]. With a mortality of up to 20% peritonitis is a dominant cause of death due to surgical infections [**1**]. 

 The causes, frequency and consequences of acute abdominal surgical emergencies due to peritonitis are different. Yet most of these are elderly patients, 60 years of age and above, with severe concomitant disorders and poor conditions. In recent decades, life expectancy has increased significantly and it may consequently be expected that demand for surgical care of the elderly is rising and will rise in the future [**2,3**].

 A prompt diagnosis and urgent surgery is life-saving for all patients with generalized secondary peritonitis [**1**]. Surgical source control is the most important determinant for survival and has to be placed on top of the therapeutic priority list. Nevertheless the therapy of sepsis requires state of the art intensive care. The mortality rate increases with the length of interval between the time of hollow organ perforation and time of surgery.[**1**]

 In this study we analyzed the demographic and clinical data of 204 patients, who underwent surgery due to generalized secondary peritonitis during one year period.

## Materials and methods

A retrospective study included one year period. A review of medical records was undertaken in order to determine patients with generalized secondary peritonitis who had undergone laparotomy at the Clinic for Emergency Surgery, between January 2009 and January 2010. A total of 204 patients undergone abdominal surgery, in the first 12 hours of admission. Included criteria were acute abdomen due to generalized secondary peritonitis as a result of perforation of any part of gastrointestinal tract. Excluded criteria were terminal phases of malignant disease and pregnancy. Demographic features, etiology, indications for surgery, intraoperative finding, morbidity and mortality were analyzed as main criteria. 

 Our approach was early and efficient surgical control of contamination, combined with effective antibiotic therapy and modern intensive care and sepsis therapy. All patients underwent exploratory laparotomy accessed through a midline incision, following a diagnosis (clinical signs and symptoms, laboratory investigations, radiological finding) of perforation -peritonitis and adequate resuscitation. After abdominal exploration, the source of contamination was sought for and controlled. The peritoneal cavity was irrigated with 4–6 liters of warm normal saline and four closed suction drains were inserted and left in abdominal cavity. In approximately 90% of patients an effective source control was achieved by one single operation with extensive peritoneal lavage. In cases with a single definitive operation, abdominal fascia was closed with continuous, number 1 non-absorbable suture. A planed staged laparotomy was necessary in only about 10% (n=20) of patients. Treatment in intensive-care unit (ICU), staged laparotomy, temporaly abdominal closure and repeated lavage was indicated in situations with: neglected peritonitis, unstable vital functions, associated life-threatening medical conditions, poor nutrition status, uncertain viability of bowel, and immunosuppression due to previous chemotherapy or radiation treatment for malignancy. All patients were given preoperative broad spectrum antibiotics, initially with a broadly calculated empirical therapy in the beginning which was later adapted to microbiological findings (from peritoneal fluid and hemoculture). 

 Statistical analysis was performed using SPSS statistical software (SPSS for Windows, release 15.0, SPSS, Chicago, IL). Descriptive statistics are presented as mean values with standard deviations for continuous variables. Categorical data are presented by absolute numbers with percentages and analyzed using the chi-square test and Fisher’s exact test. In all tests, p value<0.05 was considered to be statistically significant.

## Results

A total of 204 patients with clinical presentation of acute abdomen and generalized peritonitis, underwent surgery. The time elapsed between onset of symptoms and presentation to the hospital was less than 24 hours in 55 (26,96%) cases and more than 48 hours in 122 (59,8%) cases. The time taken for resuscitation, diagnosis and preparation of patient for surgery was less than 6 hours in 140 (68,63%) and between 6-12 hours in 64 (31,37%) patients.

 Demographic data and preoperative parameters are shown in **Table 1**. Mean age was 63,7 years (range from 3 to 90 years), 165 (80,88%) were in the age group of older than 50 years. Thirty patients were without comorbidity, 174 patients had one or more comorbidities. (**Table 1**).

**Table 1:**
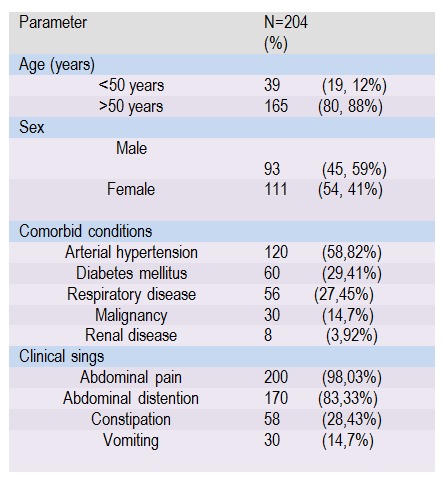
Demographics and preoperative characteristics

Etiology of peritonitis and intra-operative findings are presented in Table 2. Acid peptic disease was the most common cause of perforation peritonitis, following by the perforated appendicitis (**Table 2**). The most frequent causes of colon perforation were perforated sigmoid diverticular disease (PDD) in 28 patients (66,67%), malignancy 12 (28,57%) and inflammatory bowel disease 2 (4,76%) (IBD). Small bowel perforation due to strangulated adhesive ileus was revealed in 15 (60%) patients; in 5 (20%) patients it was caused by blund trauma, in 3 (12%) by Crohn’s disease and in 2 (8%) patients by ingested foreign bodies (chicken bone, toothpick). In 6 (2,94%) patients ruptured pyogenes tubo-ovarian abscess in females of childbearing age and in 3 (1,5%) patients liver abscess were the cause of peritonitis. In 2 (0,98%) cases we found rupture of a pyonephrotic kidney.

**Table 2:**
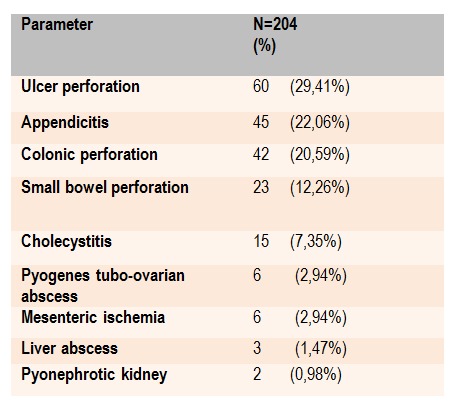
Etiology of the secondary diffuse peritonitis and intraoperative findings

**Table 3:**
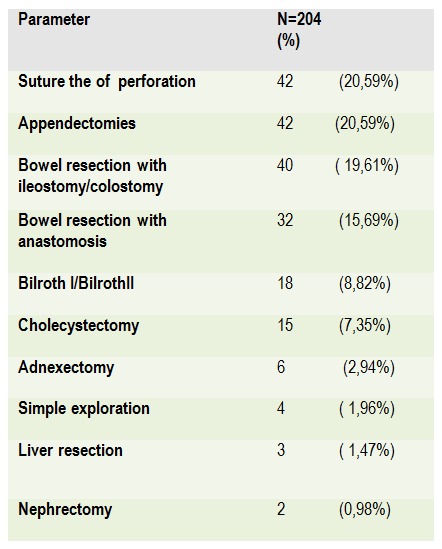
Surgical procedures in secondary diffuse peritonitis

 The surgical procedures are illustrated in **Table 3**. Peptic ulcus perforation was treated by simple suture and over sewing of the ulcer in 42 (70%) cases and gastrectomy in 18(30%). In the findings of perforated appendicitis, we performed 42 (93,33%) appendectomies and right colectomy with ileostomy was performed in 3 (6,67%) patients. In cases of colonic perforation we performed one-stage colonic resections with reconstruction in 10 (23,81%) cases, 16 (38,1%) Hartmann procedures, 12 (28,57%) right colectomy with terminal ileostomy and 4 (9,52%) subtotal colectomy with terminal ileostomy. In small bowel perforation we performed 20 (86,96%) bowel resections with primary anastomosis, 3 (13,04%) resection with ileostomy and in all cases of foreign bodies ingeston causing small bowel perforation we performed resection with anastomosis. All fifteen patients had cholecystectomy due to gallbladder perforation as a complication of the acute cholecystitis. Between 6 (2,94%) patient with mesenteric ischemia, 4 (66,67%) of them presented with complete bowel gangrene and necrosis, so operation was finished after abdominal exploration. Two cases (33,37%) with mesenteric thrombosis and necrosis of distal small bowel segments, underwent bowel resection and open mesenteric revascularization – thromboembolectomy.

Outcome following surgery due to secondary peritonitis is presented in **Table 4** and **Table 5**. The morbidity rate was 50% (n=102) (**Table 4**). Among them, 41 (40,2%) patient had a more than one complication. The morbidity rate was significantly highest among the patient with colon perforation (n=38, 90%) (Hi-square=40,1; p<0,001) (**Table 5**). Re-operation due to surgical complications was performed in 4 (1,9%) patient because of complete wound dehiscence. One patient who had previously been on radiation treatments for malignancy , underwent re-operation because of the intestinal fistula. The overall mortality rate in our study was 8,82% (n=18) (**Table 4**). The mortality rate was significantly highest among the patients with mesenteric ischemia (n=4, 66,67%) (Hi-square=45,7; p<0,001) (**Table 5**). The septicemia associated with multiple organ dysfunction syndrome (MODS) was the most common cause of death in 12 cases (66,67%) followed by respiratory complications and acute respiratory distress syndrome (ARDS) in 4 (22,23%). There was one case (5,5%) of pulmonary embolism and one acute myocardial infarction as a cause of death.

**Table 4:**
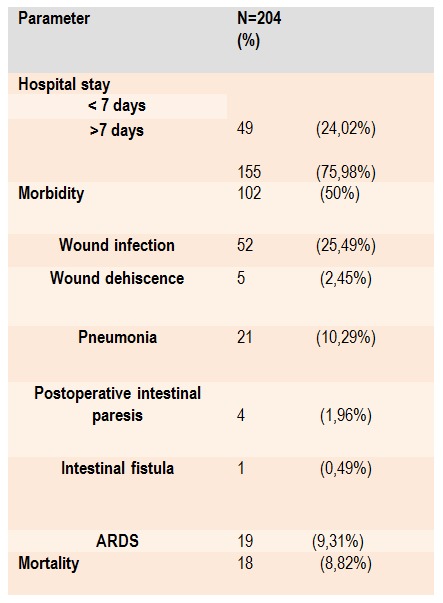
Outcome following surgery for secondary diffuse peritonitis

**Table 5:**
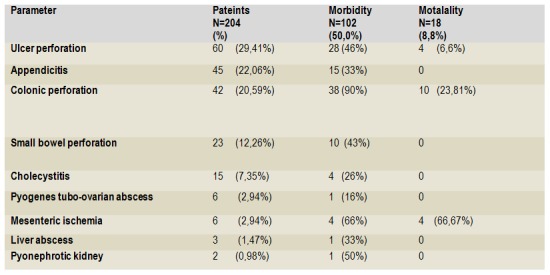
Morbidity and mortality according to ethiology of the peritonitis

## Discussion

Secondary peritonitis is common surgical emergency which presents a life-threatening condition associated with a high mortality and morbidity. Rapid source control combined with antibiotic therapy, modern intensive care and sepsis therapy are decisive for the outcome [**1**]. Intra-abdominal sepsis, although it affects all age groups, takes a greater toll on the elderly population than it does on younger populations [**4**]. The signs and symptoms are usually typical for acute abdomen and it is possible to make a rapid clinical diagnosis of peritonitis in most patients. Unfortunately, in majority of cases the presentation to the hospital is late, with well established generalized peritonitis, purulent contamination and varying degree of septicemia. A loss of physiologic reserve, together with concomitant systemic illness, results in worse outcomes for generalized peritonitis, especially in elderly population, those with imunosupresion and serious comorbid condition [**5**]. Pathophysiology of generalized peritonitis implies the complex processes in each organ system in intra-abdominal sepsis that deplete physiologic reserves and they inhibit the ability to localize, combat, and eradicate infections [**5**]. 

 This study has confirmed that the clinical presentation of the secondary peritonitis and outcome depend on duration of abdominal infection, the site of perforation and the general condition of the patient. 

 We found that patients with perforated gastroduodenal ulcers usually had a short history of pain starting in upper abdomen along with generalized abdominal tenderness. Thirty-nine (65%) of those patents had positive history of nonsteroidal anti-inflammatory drugs (NSAID) consumption. On admission 40 (66%) patients had evidence of pneumoperitoneum on abdominal X-Ray done in erect posture. These patients come to the hospital fast and dramatic symptoms and signs allow the rapid diagnosis. It is known that with the success of medical therapy, surgery has a very limited role in the management of peptic ulcer disease (PUD) today. The one of indications for urgent surgery in PUD is perforation. Many authorities recommend simple over sewing of the ulcer, with treatment of the underlying H pylori infection [**6,7**]. Additional surgical procedures for complicated PUD include vagotomy and pyloroplasty, vagotomy and antrectomy with gastroduodenal reconstruction, or a highly selective vagotomy [**8**]. 

 Acute appendicitis and appendicular perforations, addition to the typical presentation, unfortunately, may have an atypical clinical symptoms and sings. In our study among patients with appendicle perforations, 33% patients had characteristic pain, localized tenderness in right iliac fossa along with vomiting and fever. Delays in diagnosis are more common in the elderly population, in patients who are mentally confused, obese, or immunosuppressed and probably account for the higher incidences of generalized peritonitis and death [**5,9,10**]. When the diagnosis is unclear, ultrasonography (US) and in particular a computer tomography (CT) are useful [**9,10**]. The mortality rate for appendicitis in the elderly population is 2%–14%, and morbidity is 40% [**5**]. 

 Emergency surgery for acute colonic perforation is associated with a significant risk of mortality and morbidity. Colonic perforation and faecal peritonitis was observed in 20,59% patients in our study. The most frequent causes of colonic perforation were perforated sigmoid diverticular disease (PDD), malignancy and inflammatory bowel disease (IBD). We performed one-stage resections in 38% cases and 62% right or subtotal colectomy with terminal ileostomy and Hartmann procedure. 

 Colonic diverticular disease is common in Western countries, almost routinely found in elderly persons and the majority of patients (80-85%) will remain asymptomatic throughout their life [**11**]. In symptomatic cases, 0.5% will require surgery due perforated sigmoid diverticular disease (PDD) that result in generalized peritonitis [**11,13**]. Treatment guidelines for the PDD recommend Hartmann's procedure or anastomosis with protective colostomy for Hinchey stage III diverticulitis and Hartmann's procedure only for Hinchey stage IV diverticulitis [**13**]. Richter and al showed that one-stage sigmoid resection with primary anastomosis can be safe in nearly 90% of all patients with Hinchey III/IV stage [**14**]. However, in hemodynamic unstable patients, immunosuppression, feculent peritonitis with open communication with bowel lumen, and questionable viability of the bowel wall, single-stage operation is contraindication. The two-stage procedure should be used in these situations [**13**]. 

 In colorectal cancer (CRC), obstruction and perforation may occur either alone or together, at the site of the neoplasm or proximally [**15,17**]. Perforated colon perforation have a poor outcome, due to patients nutritional status, peritoneal spread of the cancer during perforation, and the emergent interventions in less prepared patients [**17**]. The common clinical presentation of colon cancer preceding perforation are abdominal pain and rectorrhagia. If the lesion is localized at or proximal to the splenic flexure, recommendation is one-stage resection and right or extended right colectomy with ileocolic anastomosis [**17**]. In the presence of lesions distal to the splenic flexure, a left hemicolectomy or anterior rectal resection is option [**16**]. In high-risk patients with septic shock, diffuse fecal peritonitis, or in the presence of unresectable cancer, alternative interventions are right or subtotal colectomy with terminal ileostomy, Hartmann procedure, bowel bypass, or colostomy [**17,18**]. 

 Postoperative adhesive disease (PAD) is not an uncommon disorder, and the incidence ranges between 12% and 17% following abdominal surgery [**19**]. PAD is common cause of the small bowel obstruction (SBO) which may lead to the bowel strangulation, ischemia and perforation [**19**]. Small bowel perforation in our study was caused by strangulation and necrosis due to adhesive ileus in mostly cases. 

 About 1–3% of patients with Crohn’s disease will present with a perforation of the intestine during their disease course [**20**]. Postoperative course in perforative Crohn's disease is often characterized by post-operative complications, anastomotic leakage and recurrent disease [**20**]. We found Crohn's disease in 3 cases of small bowel perforation, etiology of the bowel perforation was conformed on postoperative histology. After bowel resection, there was no anastomotic complications in those patient. We did not follow-up this patient more than one month. 

 Small bowel perforation caused by blunt abdominal trauma is rare but serious condition, because if the clinician does not have in mind this mechanism of bowel perforation, diagnosis may be delayed [**21**]. In this study we found 5 causes of blunt trauma caused small bowel perforation. Four injuries occurred in a traffic accident and one patient fell from a tree. All patients recovered following surgery.

 Perforation of the gallbladder is an infrequent complication of acute cholecystitis (2-15%), but it is associated with a high mortality rate without early treatment [**22,24**]. Patients with perforated gangrenous cholecystitis may have delays in presentation to the hospital, some of them presented with a benign course, with mild leukocytosis and a lack of peritoneal signs. [**24,25**] In case of complication, surgery and cholecystectomy is mandatory in 48-72 hours [**26**]. Mortality rates for gallbladder perforation decreased to 7- 16% in the following years owing to surgery, the developments in anesthesiology and intensive care conditions [**27,28**]. Laparoscopic approach is the treatment of choice for gallbladder disease, but an open approach and emergency laparotomy, cholecystectomy, lavage and drainage of the abdominal cavity is necessary in complicated cholecystitis with perforation [**22,23**]. Our study confirmed this attitude. 

 Acute mesenteric ischemia (AMI) is a life-threatening surgical emergency but a relatively uncommon cause of acute abdomen, because the mortality rates have a range of 45%–90%. [**29,30**]. Reasons are delayed diagnosis and the irreversibility of bowel ischemia after a few hours. [**30**] Risk factors for this condition include age of >50 years, cardiovascular disorders, absolute arrhythmia, and presence of multiple comorbid conditions. The causes of this serious condition are: embolus of the superior mesenteric artery (50%), thrombosis (<25%), and rare by venous thrombosis. Patients with mesenteric ischemia present with abdominal pain and sometimes bloody diarrhea, but it is important to emphasize that clinical presentation, laboratory investigations and radiological finding may all be non-specific.[**30**] Treatment includes mesenteric revascularization and resection of necrotic bowel. Unfortunately, mortality and morbidity rates after bowel resection for mesenteric ischemia are high. [**30**] In our study we performed bowel resection and mesenteric revascularization in 2 patients. In others cases of AMI, all small intestines and right colon was necrotic, so operation was finished by exploration. 

 Accidental ingestion of a foreign body occurs rarely and intestinal perforation occurs in less than 1% of ingested bodies [**31**]. The most common sites of intestinal perforation are the ileocecal and rectosigmoid regions. In our study peritonitis due to distal ileum perforation caused by foreign body was founded in 2 cases. Our approach was segmental resection of the terminal ileum and end to end anastomosis. 

 Peritonitis secondary to bilateral pyosalpinx in a young healthy woman is not very common, in those cases infection my reached the peritoneal cavity through the Fallopian tubes [**32**]. Clinical presentation is acute with signs of acute abdomen. Recommendation in acute abdomen with diffuse peritonitis is urgent laparotomy with drainage and salpingectomy [**32**].

 Rupture of a pyogenic liver abscess is relatively uncommon, reportedly occurring in 5.4% of cases [**33,34**]. The etiology of a hepatic abscess is complex, the infection my be cryptogenic in 69%, commonly in the setting of a comorbid illness such as diabetes mellitus , cardiopulmonary disease, malignancy, or liver cirrhosis [**33**]. Pyogenic liver abscesses may be caused by biliary tract disease, infection via the portal route associated with diverticulitis, pyelophlebitis, appendicitis, and proctitis; penetrating trauma and hepatic arterial microembolism in patients with sepsis [**33**]. In our study, we founded two cases of the ruptured pyogenic liver abscess in patients with diabetes mellitus, we performed debridement, pus evacuation and segment liver resection. Third patient had a perforated diverticulitis and huge liver abscess in the right lobe; surgery treatment was Hartmann procedure and liver resection.

 Peritoneal fistulization of a pyonephrosis is an extremely rare event which invariably leads to generalized peritonitis [**35**]. Rupture of a pyonephrotic kidney is usually associated with a previous kidney abnormality, renal stones and, much less commonly, neoplasms [**35**]. We revealed intraoperatively the renal origin of peritonitis, kidney was complete destroyed in both patient, patients was in severe sepsis origin in ruptured pyonephrosis.

## Conclusions

Even at the beginning of the new millennium generalized peritonitis still has a high mortality and morbidity. The atypical clinical presentation and delay in presentation can make diagnosis difficult. In addition to the availability of modern radiological methods, a careful clinical examination is still the most important way to achieve rapid, accurate diagnoses. Surgical attitude is rapid control contamination, abdominal lavage and abdominal drainage. In low risk patient, surgeons prefer a single definitive operation, but it is reasonable to use staged procedures and “second look" re-laparotomy in patients who are at high risk. There is no way to prevent peritonitis, however, efficient surgery, modern anesthesia and intensive care support my offer the chance of decreased morbidity and mortality of the intra-abdominal infections.
